# Indocyanine green fluorescence-guided TAPP for recurrent inguinal hernia: a technical note with case series

**DOI:** 10.3389/fsurg.2026.1985635

**Published:** 2026-10-21

**Authors:** Qianpeng Huang, Lihua Zhao, Jiandong Yin, An Yang, Hongbo Liu

**Affiliations:** 1 Department of General Surgery, Handan Central Hospital, Handan, Hebei Province, China; 2 Department of Nursing, Handan Central Hospital, Handan, Hebei Province, China; 3 Department of Internal Medicine, Qiu County People’s Hospital, Handan, Hebei Province, China

**Keywords:** fluorescence laparoscopy, indocyanine green, recurrent inguinal hernia, TAPP, technical note

## Abstract

**Introduction and importance:**

Laparoscopic transabdominal preperitoneal (TAPP) repair for recurrent inguinal hernias carries a nonnegligible risk of injury to the corona mortis, spermatic vessels, and inguinal nerves because of adhesions and distorted anatomy. Indocyanine green (ICG) fluorescence imaging enables real-time visualization of vascular structures, but no standardized application protocol exists for this specific setting. We describe our technique and early experience with ICG guided TAPP in recurrent hernia patients.

**Presentation of cases:**

We retrospectively analyzed 14 patients with recurrent inguinal hernia who underwent ICG fluorescenceguided TAPP between January 2023 and June 2025. All procedures were performed by a single surgeon using a 4 K fluorescence laparoscopy system. Indocyanine green (2.5 mg per dose) was administered intravenously at key steps—typically after peritoneal opening, during sac mobilization, and before mesh fixation—based on the surgeon's assessment of anatomical clarity. Fluorescence imaging was used to identify the inferior epigastric vessels, corona mortis, spermatic vessels, peri-vas deferens venous plexus, and nerve-adjacent vessels. Mean operative time was 55.9 ± 6.1 min. Mean blood loss was 7.2 ± 2.6 mL. The corona mortis was visualized in 13 of 14 cases (92.9%). No vascular injuries or chronic pain were observed during 6 month follow up. Two minor complications occurred (one seroma, one foreign body sensation).

**Clinical discussion:**

ICG fluorescence reliably identified key structures in all cases, with consistent visualization occurring 40–60s after injection. The technique was particularly valuable for delineating the corona mortis and the vascular bundles accompanying the iliohypogastric and ilioinguinal nerves, providing real-time visualization of these structures during dissection and mesh fixation. The learning curve for incorporating ICG into the standard TAPP workflow was minimal, and no ICGrelated adverse events occurred.

**Conclusion:**

ICG fluorescence is a safe and feasible adjunct in recurrent inguinal hernia TAPP. It provides real-time anatomical guidance that may assist in identifying these structures during dissection and mesh fixation. We propose a reproducible injection sequence that can be incorporated into the existing TAPP steps as a practical reference. Larger studies with longer follow-up are needed to confirm long-term outcomes.

## 1 Introduction

Recurrent inguinal hernia repair is a different operation from primary repair. The preperitoneal space, once pristine, is now occupied by scar tissue that distorts normal tissue planes and obscures anatomical landmarks (1). For surgeons performing laparoscopic transabdominal preperitoneal (TAPP) repair, the familiar road map—inferior epigastric vessels, cord structures, myopectineal orifice—becomes less reliable. Adhesions may tether the peritoneum to underlying structures, and the boundaries of the Bogros and Retzius spaces are often difficult to define (2).

The structures at risk are well documented. The corona mortis, an anastomotic connection between the inferior epigastric and obturator vessels, lies in the lateral preperitoneal space and can be encountered during dissection of the direct hernia sac or during mesh fixation in the pectineal region (3, 4). Injury to this vessel, which is present in 68% of individuals (5), can produce retroperitoneal bleeding that is difficult to control laparoscopically and may require conversion to open surgery (6). The iliohypogastric and ilioinguinal nerves, though less commonly injured in TAPP than in open repair, remain a source of postoperative chronic pain when fixation devices are placed too laterally or inferiorly (7). The spermatic vessels and vas deferens, particularly in the setting of recurrent indirect hernias, may be densely adherent to the sac, making separation hazardous.

These challenges are well recognized, yet the tools available to address them are limited. Surgeons rely on careful dissection and anatomical knowledge, but in heavily scarred fields, even experienced operators can encounter unexpected anatomy. A real-time adjunct that could make the hidden vascular and neural structures visible during dissection would be of practical value.

Indocyanine green (ICG) fluorescence imaging has been used for years in hepatobiliary surgery, colorectal surgery, and plastic surgery to visualize vascular perfusion and biliary anatomy (8–10). The principle is straightforward: intravenously injected ICG binds to plasma proteins and emits fluorescence under nearinfrared light, revealing the vascular network in real-time. Several reports have described its use in hernia surgery for assessing bowel viability in incarcerated hernias (11) and for identifying lymphatic leaks (12), but its application as an anatomical navigation tool in recurrent hernia TAPP has not been systematically described.

We began using ICG fluorescence routinely in recurrent hernia TAPP cases. Over time, we developed a standardized protocol for the timing and dosing of ICG injection. This report describes that protocol and presents our early experience with 14 consecutive patients. Where relevant, we also report our findings alongside institutional historical data from 32 patients who underwent recurrent hernia TAPP without ICG guidance, to provide descriptive context.

## 2 Methods

### 2.1 Study design and patients

This is a retrospective, single-center case series. We included all patients with recurrent inguinal hernia who underwent ICG fluorescenceguided TAPP at Handan Central Hospital between January 2023 and June 2025. During the study period, 18 patients with recurrent inguinal hernia underwent TAPP at our institution. Of these, four patients were excluded because the operating surgeon assessed that adhesions were not dense enough to require ICG guidance. The remaining 14 patients with anticipated difficult dissection due to dense adhesions were included in the final analysis. Inclusion criteria were: (1) preoperative imaging confirmation of recurrent inguinal hernia, (2) previous ipsilateral open or laparoscopic hernia repair, and (3) the operating surgeon's preoperative assessment that dissection would be difficult due to anticipated adhesions. Exclusion criteria included incarcerated hernia, simultaneous intraabdominal pathology requiring separate treatment, known ICG allergy, and severe hepatic dysfunction (Child Pugh B or C).

Fourteen patients met the criteria and formed the ICG group. For descriptive context, we also reviewed the records of 32 patients who underwent recurrent hernia TAPP before the introduction of fluorescence laparoscopy. This group served as historical reference data and was not included in the statistical analysis.

We obtained written informed consent from all patients. The study was approved by the Institutional Review Board of Handan Central Hospital (Approval No. 2023019). This report follows the PROCESS 2025 criteria (13).

### 2.2 ICG preparation and administration

ICG powder (Dandong Yichuang Pharmaceutical Co., China; 25 mg/vial) was reconstituted with 10 mL of sterile water, yielding a 2.5 mg/mL solution. Fresh solution was prepared by a trained circulating nurse immediately before the procedure and protected from ambient light until injection.

We developed a standardized three injection protocol (Figure 1).

**Figure 1:**
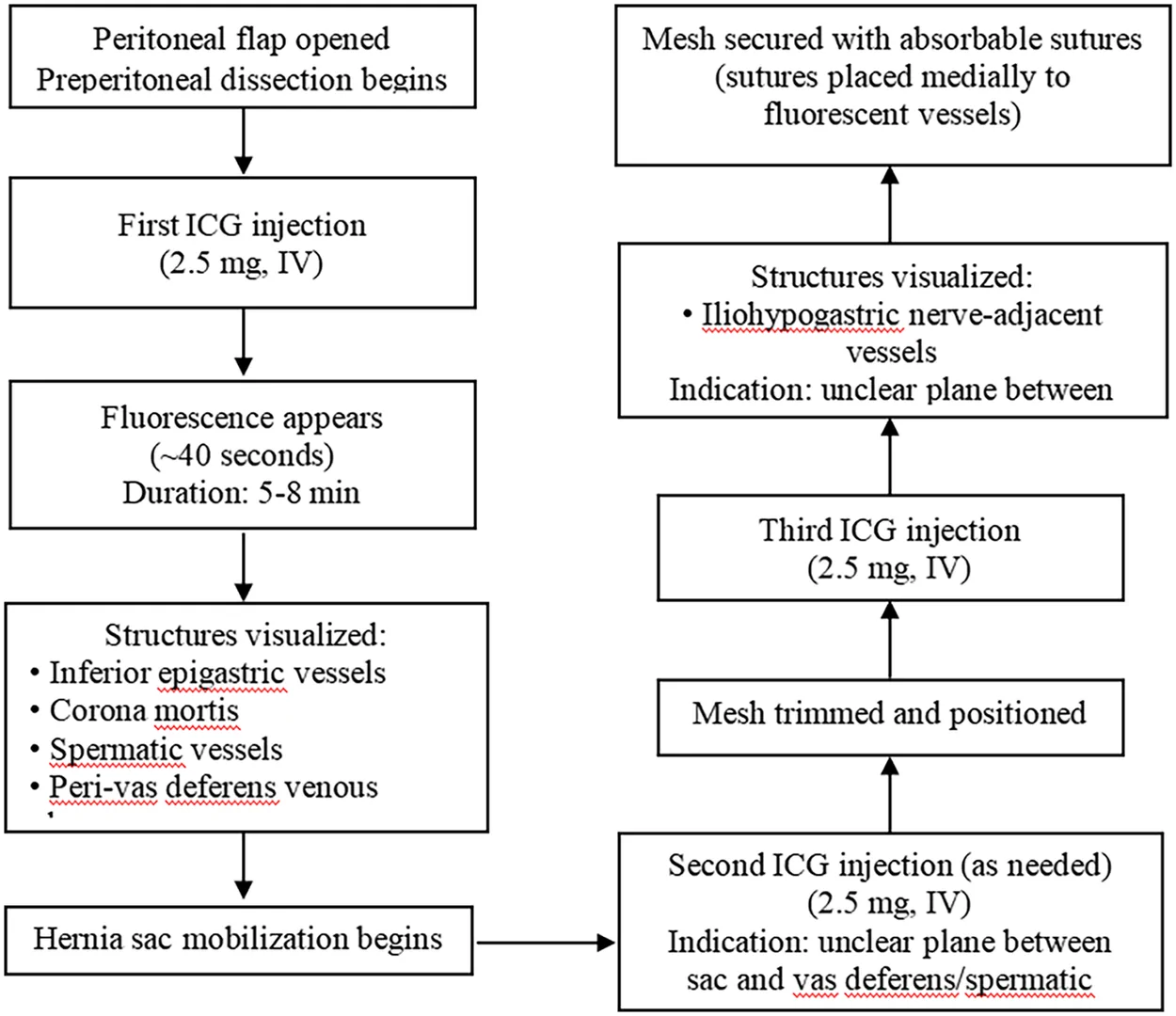
Flow diagram of ICG injection timing and anatomical structures identified at each step. (Schematic illustration of the threeinjection protocol with corresponding surgical steps and fluorescence visualization targets.).

First injection (2.5 mg): Given after the peritoneal flap is opened and the initial dissection of the preperitoneal space has begun. The purpose is to visualize the inferior epigastric vessels, corona mortis, and spermatic vessels before the dissection proceeds deeper. Fluorescence appears within approximately 40 s and lasts 4–6 min.

Second injection (2.5 mg, as needed): Given during hernia sac mobilization if the relationship between the sac, vas deferens, and spermatic vessels is unclear. This is not always required; in our series, it was used when dense adhesions around the cord made separation difficult.

Third injection (2.5 mg): Given immediately before mesh fixation, after the mesh has been trimmed and positioned. The purpose is to visualize the vascular bundles accompanying the iliohypogastric and ilioinguinal nerves, providing landmarks for safe placement of fixation devices.

Each injection was followed by a 5 mL normal saline flush through the peripheral cannula. A maximum of three injections was planned per case.

### 2.3 Surgical technique

All procedures were performed by a single surgeon using the OptoMedic 4 K fluorescence laparoscopy system (OptoMedic Co., Ltd., China), which allows toggling between white light and nearinfrared fluorescence modes. The surgeon alternated between modes during critical steps of the dissection. The historical control group had been operated on using a Storz conventional highdefinition laparoscopy system without fluorescence capability; the surgical steps were otherwise identical. The surgical steps were otherwise identical, and the same surgeon performed all procedures in both groups.

The patient was positioned supine with a slight reverse Trendelenburg tilt. Pneumoperitoneum was established at 12 mmHg through a 10 mm supraumbilical trocar. Two additional trocars (5 mm and 10 mm) were placed at the level of the umbilicus along the lateral borders of the rectus abdominis muscles.

The peritoneal flap was incised approximately 1 cm above the deep inguinal ring. The preperitoneal space was developed close to the peritoneum, extending the Bogros space laterally and the Retzius space medially. When the surgeon encountered dense fibrous tissue that obscured the underlying vessels, the first ICG injection was administered. After switching to fluorescence mode, the inferior epigastric vessels appeared as bright linear structures, and the corona mortis—if present—could be identified as a branching anastomosis in the lateral space. The spermatic vessels and the venous plexus around the vas deferens were also visible. This information guided the dissection: the surgeon could confidently divide adhesions while staying away from the fluorescent vascular structures.

When the hernia sac was encountered, the relationship between the sac and the cord structures was assessed. If the vas deferens or spermatic vessels could not be clearly separated from the sac, a second ICG injection was given. Fluorescence revealed the vessels within the cord, allowing the surgeon to identify a safe dissection plane.

After the sac was reduced and the preperitoneal dissection completed, a 10 cm × 15 cm polypropylene mesh (Rizhao Tianyi Biotechnology Medical Co., Ltd, China) was trimmed to cover the myopectineal orifice. The mesh was positioned with its inferior edge extending beyond the pubic tubercle. Before securing the mesh with sutures, the third ICG injection was given. In fluorescence mode, the small vessels accompanying the iliohypogastric and ilioinguinal nerves were visualized in the lateral preperitoneal space, allowing the surgeon to infer the course of the nerves and place the sutures accordingly. The surgeon used these as landmarks to place sutures medially to the vessels, staying outside the nervebearing zones. A 14Fr closed-suction drain was placed in the preperitoneal space. The peritoneal flap was closed with a running 3-0 absorbable suture. Trocar sites were closed in standard fashion.

### 2.4 Outcome measures

Data were extracted from electronic medical records and intraoperative video recordings. We recorded patient demographics, operative time, estimated blood loss (suction volume minus irrigation plus gauze weight difference), number of ICG injections, intraoperative complications, conversion to open surgery, postoperative drainage duration, length of hospital stay, and total hospitalization cost. Postoperative complications were recorded for seroma, hematoma, surgical site infection, mesh infection, vascular injury, chronic pain (>3 months), and sensory disturbance. Pain was assessed using a 0–10 visual analog scale (VAS) at postoperative day 1 and at 1, 3, and 6 months during follow-up. Chronic pain was defined as pain persisting beyond 3 months after surgery.

For the historical control group, the same data points were extracted from records for the 32 patients operated on before the availability of fluorescence laparoscopy. Follow-up for the ICG group was conducted at 1 week, 1 month, 3 months, and 6 months via outpatient visits or telephone interviews. Historical control patients had been followed using the same schedule.

## 3 Results

### 3.1 Patient characteristics

Fourteen patients (12 men, 2 women) with a mean age of 59.9 ± 11.6 years were included in the ICG group. Ten patients had previous open repair without mesh, one had a previous Lichtenstein repair, and three had previous TAPP repairs. The median interval between primary and recurrent repair was 11.0 months (IQR: 8.5–14.5). Nine recurrences were on the left side and five on the right. Ten hernias were direct and four indirect according to the EHS classification. Baseline characteristics are shown in Table 1.

**Table 1:** Baseline characteristics of the ICG group (*n* = 14).

Characteristics	ICG cohort (*n* = 14)
Age (years)	59.86 ± 11.64
Sex, *n* (%)	
Male	12 (85.7)
Female	2 (14.3)
BMI (kg/m^2^)	23.84 ± 1.71
Time since previous surgery (Year)	11.0 (8.5, 14.5)
Previous surgical approach, *n* (%)	
Mesh placement	4 (28.6)
Without mesh	10 (71.4)
Side of recurrence, *n* (%)	
Left	9 (64.3)
Right	5 (35.7)
Type of recurrence, *n* (%)	
Indirect hernia	4 (28.6)
Direct hernia	10 (71.4)
Internal ring classification (EHS), *n* (%)	
I	1 (7.1)
II	10 (71.4)
III	3 (21.4)

### 3.2 ICG fluorescence imaging

All 14 patients received ICG injections. In all cases, fluorescence was observed within 40–60 s after injection and persisted for 4–6 min. In five cases (35.7%), the initial fluorescence signal faded before the completion of cord structure mobilization, and given the dense local adhesions, a second injection was administered to extend the visualization time and avoid injury to the vas deferens and spermatic vessels. In the remaining nine cases (64.3%), the first injection successfully delineated the location of the critical vessels. As the local adhesions were relatively mild, the intended dissection steps could be safely completed without the need for real-time fluorescence guidance. The inferior epigastric vessels and spermatic vessels were identified in all 14 cases (100%). The corona mortis was identified in 13 cases (92.9%); in one case, no anastomotic vessel was visible, suggesting either anatomical absence or a vessel too small to generate detectable fluorescence. The vascular bundles accompanying the iliohypogastric and ilioinguinal nerves were identified in 11 of 14 cases (78.6%) following the third injection. In the remaining three cases, the vessels were not clearly visualized, possibly due to patient-related factors such as low cardiac output or a small caliber of the vessels. Figure 2 illustrates these intraoperative fluorescence findings, including the inferior epigastric vein (red arrow), corona mortis (blue arrow), peri-vas deferens venous plexus (green arrow), spermatic venous plexus (yellow arrow), and nerve-adjacent vessels (black arrow).

**Figure 2:**
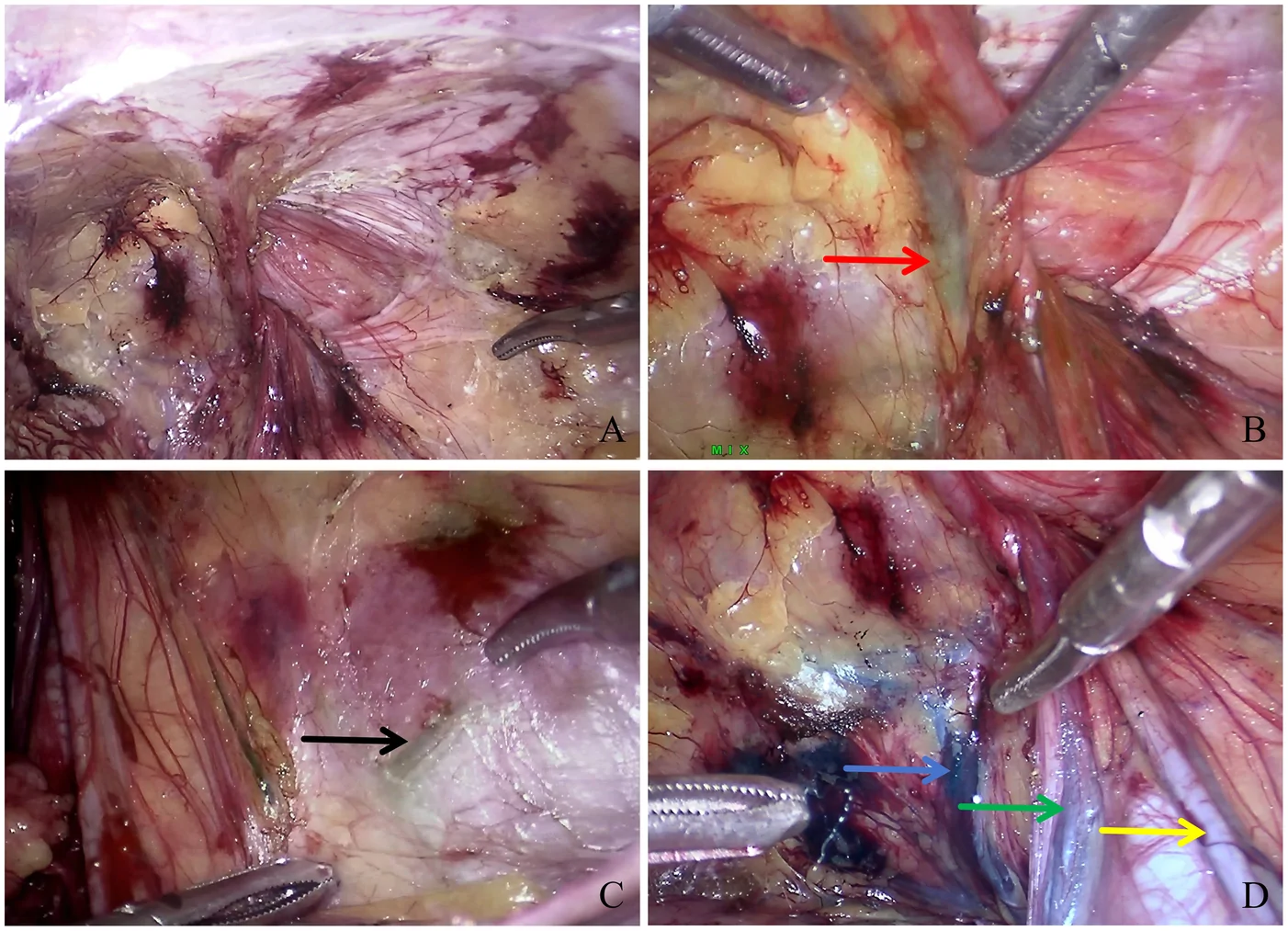
Intraoperative fluorescence images during ICG-guided TAPP. **(A)** White-light view of the preperitoneal space showing dense adhesions obscuring the anatomy. **(B)** Fluorescence view showing the inferior epigastric vein (red arrow). **(C)** Fluorescence view showing the nerve-adjacent vessels (black arrow). **(D)** Fluorescence view showing the corona mortis (blue arrow), the peri-vas deferens venous plexus (green arrow), and the spermatic venous plexus (yellow arrow).

In all 14 cases, the mesh was secured with absorbable sutures. Before fixation, the third ICG injection was given to visualize the small vessels accompanying the iliohypogastric and ilioinguinal nerves. The surgeon placed the sutures medially to these fluorescent vessels, using them as landmarks to avoid the nerve-bearing zones. No ICG related adverse events were observed. None of the patients had allergic reactions, hypotension, or changes in liver function tests postoperatively.

### 3.3 Representative case illustration

A 62 year old man with a left recurrent indirect hernia was referred to our service. He had undergone a Bassini repair 14 months earlier. Preoperative examination revealed a reducible bulge in the left groin, and CT showed a recurrent hernia with extensive preperitoneal scarring.

During TAPP, after opening the peritoneal flap, the preperitoneal space was densely scarred. The inferior epigastric vessels were difficult to identify in white light mode. After the first ICG injection (2.5 mg), fluorescence revealed the inferior epigastric vein and its branches. More importantly, a branching vascular structure was observed in the lateral preperitoneal space—the corona mortis—which was not visible before ICG. The surgeon dissected around this structure, preserving it intact.

When mobilizing the hernia sac, the vas deferens was densely adherent to the sac. A second ICG injection clarified the plane between the sac and the spermatic vessels, allowing the surgeon to separate them without injury.

After mesh placement, a third ICG injection revealed small vessels accompanying the iliohypogastric nerve. The surgeon placed two absorbable sutures medially to these vessels, using the fluorescent vessels as landmarks to avoid the nerve. The operation took 58 min, with 10 mL estimated blood loss. The patient was discharged on postoperative day 3. At 6-month follow-up, he had no pain, no numbness, and no recurrence.

### 3.4 Surgical outcomes in the ICG group

All 14 procedures were completed laparoscopically without conversion. Mean operative time was 55.9 ± 6.1 min (range: 45–67 min). Mean estimated blood loss was 7.2 ± 2.6 mL (range: 3–12 mL). Mean drainage duration was 1.7 ± 0.7 days, and mean hospital stay was 4.0 ± 0.8 days. Total hospitalization cost averaged 8,717 ± 518 RMB. Detailed perioperative outcomes are presented in Table 2.

**Table 2:** Perioperative outcomes and postoperative complications in the ICG group (*n* = 14).

Parameters	ICG cohort (*n* = 14)
ASA score	1.14 ± 0.36
Operative time (min)	55.93 ± 6.09
Estimated blood loss (mL)	7.21 ± 2.58
VAS score (postoperative day 1)	2.57 ± 1.45
Drainage duration (days)	1.71 ± 0.73
Total hospitalization cost (RMB)	8,717.14 ± 518.01
Complications, *n* (%)	
Mesh infection	0
Seroma	1 (7.1)
Hematoma	0
Recurrence	0
Chronic pain	0
Foreign body sensation	1 (7.1)
Vascular injury	0
Sensory disturbance	0
Total complications	2 (14.3)

### 3.5 Postoperative complications and follow-up in the ICG group

Two patients developed minor complications (14.3%). One patient had a small seroma at the dissection site that resolved spontaneously within 2 weeks. One patient reported a foreign body sensation in the left groin, which gradually diminished by the 3month visit. No patient developed hematoma, surgical site infection, mesh infection, vascular injury, or sensory disturbance, and no patient reported chronic pain at the 3- or 6-month follow-up visits. No recurrence was detected during the 6month followup period. Complication data are summarized in Table 2.

## 4 Clinical discussion

This case series describes a standardized protocol for using ICG fluorescence imaging during TAPP repair of recurrent inguinal hernias. Four points warrant discussion: the practical utility of the technique, the practical challenges encountered, the standardized injection protocol, the contextual reference to historical data, and the limitations of our experience.

Practical utility of ICG in recurrent hernia TAPP. The central difficulty in recurrent hernia repair is that the usual anatomical cues are no longer reliable. Scar tissue can obscure the inferior epigastric vessels, and the corona mortis—a structure that many surgeons prefer to avoid rather than identify—can be encountered unexpectedly during lateral dissection (4). In our series, ICG reliably visualized these vessels in 92.9%–100% of cases, with fluorescence appearing within a predictable time window after injection. This provided the surgeon with real-time information about the location of these vessels before dissection.

The nerve-adjacent vessels proved particularly useful for mesh fixation. The iliohypogastric and ilioinguinal nerves are not directly visualized in TAPP, but their general location can be inferred from the vascular bundles that accompany them. By placing tackers medially to these fluorescent vessels, the surgeon avoided the typical nerve zones. No chronic pain was observed in this small series during the 6-month follow-up; however, this finding should be interpreted with caution given the limited sample size and short follow-up (14).

Practical challenges and technical observations. We encountered no ICGrelated adverse events, but technical challenges were noted in a few cases. In one patient, the corona mortis was not visualized despite adequate ICG injection and normal fluorescence of adjacent vessels, suggesting either anatomical absence or a vessel caliber below the detection threshold of the imaging system. In two patients, the fluorescence signal was subjectively weaker than expected, possibly related to lower cardiac output during anesthesia; in these cases, repeating the injection after adjusting the patient's hemodynamic status improved visualization. We also observed that the optimal imaging window—approximately 40–60 s after injection—was relatively brief; the surgeon must be ready to toggle to fluorescence mode promptly to capture the signal. These observations underscore that ICG is an adjunct to, not a substitute for, anatomical knowledge and careful dissection.

Standardization of the injection protocol. One of the goals of this report was to propose a protocol that could be replicated by other surgeons. We found that three injections at predefined steps provided adequate guidance without excessive dosing. The first injection, given after peritoneal opening, maps the major vessels in the preperitoneal space. The second, used selectively during sac mobilization, clarifies the cord structures in difficult cases. The third, before mesh fixation, marks the safe zones for tacker placement. This sequence fits into the existing TAPP workflow without adding significant time. The mean operative time in our series (55.9 min) falls within the lower range of the 60–90 min commonly cited in the literature (15, 16).

Contextual reference to institutional historical data. For descriptive context, we reviewed the records of 32 patients who underwent recurrent hernia TAPP at our institution prior to the availability of fluorescence laparoscopy. In that historical cohort, the mean operative time was 66.9 ± 6.1 min, mean estimated blood loss was 17.8 ± 12.6 mL, and the overall complication rate was 46.9% (15/32). Although no statistical comparisons were performed between the two groups due to the non-randomized and non-contemporaneous design, the descriptive data provide context for interpreting our preliminary findings. It should be noted that the historical cohort was operated on using a conventional high-definition laparoscopy system without fluorescence capability. These factors may have contributed to the observed differences, and the historical data should therefore be interpreted as descriptive context only, not as a direct comparison. However, these findings should be interpreted with caution given the inherent differences in surgical era and patient selection, and a prospective comparative study is warranted to validate these results.

Comparison with prior literature. To our knowledge, this is the first description of ICG fluorescence as a systematic anatomical navigation tool in recurrent inguinal hernia TAPP. Previous studies have used ICG in hernia surgery for other purposes: assessing bowel viability in incarcerated hernias (11), identifying lymphatic leaks after open repair (12), and evaluating perfusion of the inguinal skin flap in complex abdominal wall reconstruction (17). None have described its use for intraoperative guidance of dissection and mesh fixation in recurrent hernias. Our report fills that gap by presenting a practical protocol and documenting its feasibility.

### 4.1 Limitations

This is a small, single-center case series. The ICG group had no contemporaneous control, and the historical reference data are subject to selection bias and temporal confounders. The historical cohort was operated using a conventional high-definition laparoscopy system, whereas the ICG group benefited from both the learning curve and a 4 K fluorescence system. These factors, along with the selection of patients with anticipated dense adhesions, may have contributed to the observed differences and preclude any direct attribution to ICG alone. The 6-month follow-up is insufficient to draw conclusions about long-term recurrence rates, and the absence of chronic pain should be interpreted with caution given this limited follow-up. Additionally, the technique is not generalizable to institutions without fluorescence laparoscopy. We are continuing to collect data and plan to extend follow-up to 2 years.

## 5 Conclusion

ICG fluorescence imaging is a safe and feasible adjunct in TAPP repair of recurrent inguinal hernias. The standardized protocol described here—three injections timed to coincide with the key steps of dissection—integrates smoothly into the existing surgical workflow. The technique provides real-time visualization of the corona mortis, spermatic vessels, and nerve-adjacent vessels, structures that are often obscured in the scarred preperitoneal space. This anatomical guidance may assist in identifying these structures during dissection and mesh fixation. Larger studies with longer follow-up are warranted to confirm these preliminary findings.

## Data Availability

The datasets presented in this study can be found in online repositories. The names of the repository/repositories and accession number(s) can be found in the article/Supplementary Material.
