# Supplementary material for: Indocyanine green fluorescence-guided TAPP for recurrent inguinal hernia: a technical note with case series
Source: Front Surg. 2026 Oct 21;13:1985635. doi: 10.3389/fsurg.2026.1985635 (PMC13732770; doi:10.3389/fsurg.2026.1985635)
Supplement: Supplementary file 1 [file Table1.docx]

Supplementary Table 1. Perioperative outcomes of the historical control group

| Perioperative outcomes | Historical cohort (n=32) |
| --- | --- |
| Operative time (min) | 66.94±6.09 |
| Estimated blood loss (mL) | 17.78±12.56 |
| Total complications | 15 (46.9) |
